# Oxidative stress and Rho GTPases in the biogenesis of tunnelling nanotubes: implications in disease and therapy

**DOI:** 10.1007/s00018-021-04040-0

**Published:** 2021-12-18

**Authors:** Abinaya Raghavan, Pooja Rao, Jiri Neuzil, Dean L. Pountney, Sangeeta Nath

**Affiliations:** 1 Manipal Institute of Regenerative Medicine, Bangalore, Manipal Academy of Higher Education, Manipal, 560065 India; 2 School of Pharmacy and Medical Science, Griffith University, Southport, QLD 4222 Australia; 3 Institute of Biotechnology, Czech Academy of Sciences, 252 50 Prague-West, Czech Republic

**Keywords:** Intercellular transfer, Mitochondrial homeostasis, Reactive oxygen species (ROS), Apoptosis, Cellular stress, Chemotherapy resistance, Mesenchymal stem cells, Rejuvenation

## Abstract

Tunnelling nanotubes (TNTs) are an emerging route of long-range intercellular communication that mediate cell-to-cell exchange of cargo and organelles and contribute to maintaining cellular homeostasis by balancing diverse cellular stresses. Besides their role in intercellular communication, TNTs are implicated in several ways in health and disease. Transfer of pathogenic molecules or structures via TNTs can promote the progression of neurodegenerative diseases, cancer malignancy, and the spread of viral infection. Additionally, TNTs contribute to acquiring resistance to cancer therapy, probably via their ability to rescue cells by ameliorating various pathological stresses, such as oxidative stress, reactive oxygen species (ROS), mitochondrial dysfunction, and apoptotic stress. Moreover, mesenchymal stem cells play a crucial role in the rejuvenation of targeted cells with mitochondrial heteroplasmy and oxidative stress by transferring healthy mitochondria through TNTs. Recent research has focussed on uncovering the key regulatory molecules involved in the biogenesis of TNTs. However further work will be required to provide detailed understanding of TNT regulation. In this review, we discuss possible associations with Rho GTPases linked to oxidative stress and apoptotic signals in biogenesis pathways of TNTs and summarize how intercellular trafficking of cargo and organelles, including mitochondria, via TNTs plays a crucial role in disease progression and also in rejuvenation/therapy.

## Introduction

Cell-to-cell communication plays an important role in maintaining tissue homeostasis. Intercellular communication can be facilitated by many soluble factors such as growth factors, neurotransmitters, cytokines, and extracellular vesicles (EVs), such as exosomes. A study in 2004 [1], first described intercellular transfer of molecular information directly between distal cells forming f-actin containing membrane lipid bilayer encircled ‘tunnel’ structures. Since then, the term “tunnelling nanotube” (TNT) has referred to this membrane f-actin conduit. Originally, the diameter of TNTs was reported to be 50–200 nm [1]. Later studies reported a relatively thicker diameter of around 700–900 nm, using optical resolution limited methods [2]. Cancer cells form networks of TNT-like but relatively thicker membrane protrusions, termed as tumour microtubes (TMs), consisting of both f-actin and tubulin. They are closed-ended and connected via gap junctions at the ends to transfer electrical signals and small molecules [3, 4]. Several studies have also referred to thinner nano-scaled membrane actin closed-ended protrusions as TNTs. Conventionally, f-actin containing open ended nanostructures are termed as TNTs. Recently, correlative FIB-SEM, light- and cryo-electron microscopy of neuronal cells revealed that TNTs of diameter 550 nm are made of 2–11 bundles of thinner channels (iTNTs), where the average diameter of each iTNTs was 123 ± 66 nm [5]. TNTs allow for the intercellular transport of various cargos, including viruses, organelles, RNAs, proteins, and toxic materials such as neurodegenerative protein aggregates [6]. Transfer of mitochondria has been implicated in disease progression and also in regeneration. Several studies have shown that intracellular build-up of prions or prion-like proteins facilitate disease progression by transferring toxic aggregates of these proteins or stressed organelles such as lysosomes and mitochondria from pathological donor cells to healthier acceptor cells [7, 8]. On the other hand, healthy mitochondria from mesenchymal stem cells (MSCs) are transferred to targeted acceptor cells with non-functional mtDNA/mitochondria [6, 9, 10]

In addition to mediating intercellular communication, TNTs rescue cells by relieving diverse cellular stresses caused by pathological conditions, such as oxidative stress, reactive oxygen species (ROS), mitochondrial heteroplasmy and apoptotic stress [11, 12]. Although the molecular drivers for the formation of TNTs under various pathophysiological conditions are unclear, studies over the last two decades indicate that cells form direct long-range connections between neighbouring cells via TNTs to alleviate cellular stress. Cytoskeletal dynamics play a pivotal role in the formation of TNTs and several studies have implicated the localized control of Rho GTPases in TNT-linked actin polymerization pathways [3, 13]. It has become evident that classical Rho GTPases (Rac1, Cdc42, and RhoA) control the complex regulatory balance in cell cycle progression and apoptotic signalling pathways [13, 14]. The capacity of MSCs to donate healthy mitochondria to targeted acceptor cells via TNTs correlates with the activity and expression of the atypical mitochondrial Rho GTPases [15], Miro-1 [9] and Miro-2 [16]. In this review, we summarize the role of TNTs in counteracting oxidative stress, mitochondrial heteroplasmy and apoptosis-related diverse cellular stresses, and the possible association of Rho GTPase-linked apoptotic signalling pathways in cytoskeleton remodelling and plasma membrane surface dynamics in the biogenesis of TNTs.

## TNTs in intercellular transport

The original report [1], showed the transfer of endocytic vesicles and organelles as intercellular mediators between pheochromocytoma (PC12) cells. Subsequently, several studies in various cellular systems have shown the presence of TNTs and a range of organelles and cargo transportation via TNTs. These cargos include cytosolic proteins [17], ions [18], and miRNAs [19] that propagate between cells.

Various cellular stresses and pathological conditions promote intercellular transfer of organelles including the endoplasmic reticulum, golgi [12], mitochondria [20], endosomes [21] and lysosomes [7] via TNTs. Transfer of lysosomes from healthy endothelial progenitor cells to stressed human umbilical vein endothelial cells (HUVEC) has been reported, and this transfer helps to maintain lysosomal pH [22]. Oxidative stress-induced transfer of aberrant mitochondria via TNTs helps to propagate pathology from stressed to healthy cells in several diseases [23]. On the other hand, the transfers of healthy mitochondria from MSCs to targeted stressed cells is emerging as a potential therapy in regeneration [9, 10, 24, 25]

## TNTs in the spread of disease pathology

Studies in 2005–2010 reported the transfer of prion proteins [26], bacteria [27], and viruses [28] from cell to cell through nanotubes leading to the spread of pathology. Viruses such as human immunodeficiency virus (HIV), and herpesviruses use this intercellular mode of dissemination without exposing themselves to the extracellular environment, thereby escaping the humoral immunity of the host [29, 30]. The first report about the propagation of virus particles from infected to uninfected T cells via TNTs was described for HIV [28]. Later, the involvement of TNTs in the spread of viruses has also been demonstrated for the influenza A virus [31], DNA viruses including alpha herpesvirus [32], bovine herpesvirus 1 [33] and human T-cell leukemia virus type 1 [34].

Initial studies demonstrated in 2009 that prions can hijack TNTs to spread the prion pathology in a cell-to-cell manner [26]. Subsequently, the intercellular propagation of amyloidogenic proteins via TNTs has been widely studied. Several such studies have demonstrated the spread of neurodegenerative proteins such as α-synuclein [35, 36], tau [37, 38], amyloid β [12, 39] and huntingtin [40]. One of the major hallmarks of neurodegenerative diseases is insufficient degradative capacity of lysosomes due to the accumulation of proteotoxic aggregates [41, 42], and lysosomal accumulation generates mitochondrial toxicity and increased oxidative stress. Evidence from several studies indicates that lysosomes can mediate the spread of neurodegenerative protein aggregates via TNTs [7]. It has also been demonstrated that α-synuclein aggregates can be transferred from cell to cell bound to mitochondria travelling within TNTs between neuronal cells [8].

## TNTs in cancer malignancy

TNTs that are formed between malignant cells or between malignant cells and other cells in the tumour matrix are known to initiate tumour formation and metastasis [43]. Cell-to-cell transfer of mitochondria via TNTs plays a crucial role in maintaining metabolic homeostasis in cancer cells [44]. Below we discuss several key reports regarding cancer malignancy and TNTs.

Tumour cells network via nano-sized actin membrane open-ended conduits (TNTs proper) or by relatively thicker closed-ended micro-sized tubes (TMs) containing tubulin to transport organelles. The initial study [45], first demonstrated TNT like structures in intact solid tumours dissected from patients with lung adenocarcinoma and pleural mesothelioma malignant tissues. More recently, tumour cell-derived networks of membrane-tubes were observed in animal models of astrocytic brain tumours, including glioblastomas (GBM tumours) [46]. The structures are longer and thicker in diameter, and referred to as TMs. Intercellular transfer of mitochondria from tumour-activated stromal cells (TASC) by means of TNTs, EVs or cannibalism promotes proliferation of patient derived primary cultures of GBM cells in a 3D environment [47]. GBM stem-like cells (GSLCs) used in 2D culture and 3D organoid culture showed mitochondrial transfer via TNTs. These studies proposed a role of TNTs and TMs in the context of malignancy spread in organoid tumour models [48].

Mitochondrial transfer by means of TNTs from non-malignant bone marrow stromal cells to multiple myeloma cells resulted in tumour progression [49]. The same study also showed that shRNA-mediated CD38 knockdown inhibited mitochondrial transfer in vivo. The same knockdown in the in vivo model resulted in attenuation of tumour growth and improved survival rate of animal. In addition, hypoxia elevated the formation of TNTs and malignancy in ovarian and colon cancer [50]. This state of oxygen insufficiency results in increased levels of ROS in tumour cells, which leads to increased metabolic rate, gene expression, mitochondrial peroxidation, cellular stress and apoptotic stress [51, 52]. Cancer cells can counteract ROS induced apoptosis by enzymatic and non-enzymatic antioxidant defences, and it is now well accepted that moderate levels of ROS contributes to tumour progression by promoting several signalling pathways and gene mutations [53]. Several recent studies have shown that ROS promotes formation of TNTs and TNTs contribute in developing malignancy and resistance to cancer therapy [54].

Bcl-2, a highly conserved anti-apoptotic protein plays a central role in acquiring resistance to cancer therapy. A recent study [55] has shown that TNTs contribute to the progression of colorectal cancer by upregulating ERK (extracellular signal regulated kinase) expression in recipient cells by transferring mutant KRAS to these cells. They tend to develop TNTs as a part of their invasion and migration processes, and to transfer miRNAs as regulators of signalling pathways [56–58]. All these recent reports and several other studies (summarized in the Table 1) document that TNT formation is directly related to tumour malignancy and plays a significant role in tumour adaptation.

**Table 1:** Transfer of pathology spreading through TNTs in cancer malignancies and acquiring of cancer therapy resistance

Type of cell	Type of study	Movement of mitochondria	Result
Rat PC12 derived from Pheochromocytoma	In vitro	A two-way motion of mitochondrial movement was observed in the healthy cells whereas in unhealthy cells the mitochondrial movement was unidirectional (healthy-unhealthy)	The UV-treated cells were retrieved [25]
MSCs, cisplatin treated NSCs	In vitro	Mitochondria transfer from MSCs to cisplatin induced NSCs	Survival of cisplatin induced NSCs [59]
Patient derived primary Glioblastoma stem cells (2D and 3D)	In vitro	Transfer of mitochondria from glioblastoma stem cells to tumour organoid	The transfer of mitochondria was observed after the irradiation treatment [56]
Human tumour activated stromal cells (TASCs) and glioblastoma cells	In vitro	The transfer occurred from TASCs to glioblastoma cells in 3D/organoid condition	The proliferation of GBM cells occurred along with chemoresistance [47]
U87 glioblastoma cells and chemo resistance U87^RETO^ cells	In vitro	Cytotoxic stress by etoposide	Accumulation of mitochondria in chemo resistance cancer cells [60]
Multiple myeloma primary cells(human) and cell lines, bone marrow stromal cells (BMSC)	In vitro	The transmission occurred between BMSC and myeloma cells	The proliferation increased in myeloma cells and higher ATP production [49]
	In vivo	Knockdown of CD38 inhibits transfer of mitochondria	CD38 knockdown in animal model improves their survival by inhibiting myeloma growth [49]
Human AML (acute myeloid leukemia) blasts and BMSCs	In vitro	NOX2 induced superoxide promotes the transfer between BMSC to AML cells	Greater basal and highest mitochondrial respiration and ATP production was observed in AML cells [61]
	In vivo	Inhibition of NOX2 prevents transfer in AML mouse	Apoptosis in AML and improved survival of AML mouse [61]
Primary cells derived from human malignant mesothelioma, mesothelioma cell lines and healthy mesothelial cells	In vitro	The transfer occurred between malignant cells / among the healthy cells, but not between cancer cells and normal cells	Cancer cell etiology and conquest [45]
Human prostatic cancer cells (PCa), cancer-associated fibroblasts (CAFs)	In vitro	The transfer ensued from CAFs to PCa cancer cells	A higher migratory and metastatic capacities of PC3 cancer cells were observed [62]
	In vivo	Tumour growth and transfer was observed in PCa tumour models	
Mesenchymal stem cells, ECs, ovarian cancer cell line and breast cancer cell line	In vitro	A two-directional movement was seen	Chemoresistance was observed [63]
Human T24 urothelial carcinoma cells and non-malignant urinary papillary urothelial cell (RT4)	In vitro	The transfer happened between malignant to non-malignant cells	Increased non-malignant cell intrusiveness [64]
	In vivo	Increased invasiveness of bladder cancer cells	
MSCs and acute lymphoblastic leukemia (ALL) cells	In vitro	It occurred from chemotherapy activated MSCs to ALL cells	Chemoprotection occurred by the ROS-induced pathway [65]
	In vivo	Chemotherapy activated MSCs disseminated mitochondria to ALL cells in murine NSG model	
MSCs, Jurkat cells and T-ALL cells	In vitro	A mutual exchange occurred between the Human MSCs, Jurkat cells and T-ALL cells	Jurkat and T-ALL cells developed chemoresistance [66]
Wharton jelly mMSCs and osteosarcoma cells	In vitro	mtDNA deleted osteosarcoma (143 ρ^0^ cells)	OXYPHOS dependent cell proliferation and restoration of bioenergetics [67]
Senescent primary human fibroblast line HF043	In vitro	The transfer happened between senescent cells	mTOR and Cdc-42 signalling pathways involve in TNT formation [68]
Chemoresistant and chemosensitive ovarian cancer cells	In vitro	Hypoxia	Cancer cells were synchronized against chemotherapy [69]

## TNTs in drug resistance

Intercellular communications were suggested as a potential target for anti-cancer therapies as early as 2004 [70]. Several recent studies have demonstrated that TNT and TM networks play crucial roles in making these tumours exceptionally resistant to therapy [48]. Mitochondrial transfer from tumour activated stromal cells (TASC) to glioblastoma (GBM) cells was observed via TNTs, and the process provided chemo- and radio-resistance of the GBM [47]. Another study around the same time showed, GBM cells import the DNA repair enzyme O6-methylguanine-DNA methyltransferase via TNTs, thus enhancing resistance to temozolomide [71]. A self-repair mechanism of laser irradiated brain tumour cells was observed, and it involved formation of a network of TNTs and TMs [46]. Furthermore, GBM cells irradiated with α- particles establish a network of TNTs more rapidly compared to control irradiated cells in vitro within 24 h [72].

TNT-mediated cancer drug resistance and rescue from apoptotic cell death is a great challenge in cancer treatment. Acquisition of mitochondria in cancer cells (MCF-7) from endothelial cells through TNTs resulted in doxorubicin resistance in MCF-7 cells [63]. Later, in 2015 [73], it was shown that disruption of TNTs decreased the resistance of B-cell precursor acute lymphoblastic leukemia (BCP-ALL) cell to antileukemic drug prednisolone. A study in pancreatic cancer cells showed, doxorubicin increased the formation of TNTs in vitro in a dose-dependent manner and the biogenesis of TNTs promotes resistance to chemotherapy. The observation of drug resistance was also demonstrated in vivo in tumour specimens from patients diagnosed with pancreatic adenocarcinoma and neuroendocrine carcinoma [74]. The study by Wang et al. [66], showed that mitochondrial exchange through TNTs from Jurkat cells to MSCs by ICAM-1 mediated cell adhesion led to chemoresistance (Ara C and Methotrexate) in Jurkat cells. They also showed inhibition of TNT formation led to reduced chemoresistance in primary T-ALL cells (T cell acute lymphoblastic leukemia). Chemotherapy drugs, cytarabine (Ara-C) and doxorubicin (DNR), activated MSCs to disseminate mitochondria to surrounding ALL cells, and as a result chemoresistance developed [65]. Moreover, transfer of myosin containing cellular vesicles from stromal cells to chronic myeloid leukemia cells resulted in increased resistance of leukemic cells to imatinib which is a tyrosine kinase inhibitor [75].

## Mitochondrial transfer from mesenchymal stem cells via TNTs

From a therapeutic point of view, TNTs can play a significant role in stem cell therapy, while the same cellular processes can be detrimental in certain pathological conditions. Several studies have shown that the transfer of mitochondria primarily depends on the communication between MSCs and target cells, and this communication is governed by several mechanisms. They include EVs, gap junctions, cell fusions, and TNTs [76]. Mitochondria provide the capacity for aerobic respiration, play important roles in aging and dysfunction in various heritable and acquired diseases. The human mitochondrial genome has 16,568 bp and encodes for only a small set of mitochondria-specific proteins, rRNAs and tRNAs, while majority of proteins are encoded by the nucleus [77]. The mutation rate in the mtDNA genome is high because it is not protected by histones and has low-efficiency nucleotide repair mechanisms [78].The first report of mitochondrial transfer from MSCs was published in 2006, and showed rescue of aerobic respiration by transferring functioning mitochondria via TNTs to cancer cells devoid of mtDNA [10]. Following this, several studies reported a high propensity of mitochondrial propagation and dynamics through TNTs extended from MSCs to the targeted somatic cells [20, 79–81]. Researchers have documented transfer of mitochondria from MSCs to the HUVEC, which are initially subject to ischemia–reperfusion injury [82]. A study in a mouse model of lung injury showed transfer of mitochondria from bone marrow-derived stromal cells to pulmonary alveoli caused alleviation of respiratory damage [83].

Recent research has shown that MSCs from tissue of divers origins, such as bone marrow, Wharton's jelly, adipose, and dental pulp play a role in protecting damaged cells from oxidative stress by donating mitochondria [84]. Studies have also demonstrated that MSCs play a crucial role in reducing mitochondrial ROS levels during repair pathways [9, 85]. However, it is not clear why MSCs exclusively form TNTs to targeted cells and what signal stimulates healthy MSCs to induce TNTs and transfer functional mitochondria. Paracrine factors released from neighbouring stressed cells modulate MSCs to initiate its action of damage repair. One study has shown that phosphatidylserine externalized on the surface of damaged cells (apoptotic epithelial cell) prompted MSCs to form TNTs [80]. In another study, it has been shown that connexin 43 plays a vital role in the regulation of TNT formation [86]. The same study has shown that iPSC derived MSCs transfer mitochondria via TNTs to rescue injured lung epithelial cells in a mouse model as well as in an in vitro model. This “donation” of mitochondria helped in alleviating asthma-related inflammation levels due to hypoxic conditions, and also prevented apoptosis of epithelial cells. One study [87] has shown that transfer of mitochondria via TNTs from MSCs to ocular cells helped in increasing the aerobic capacity and upregulation of mitochondrial genes. The work [88] suggested that both paracrine factors and mitochondrial transfer protect cardiomyocytes against stress, independent of each other.

In the last 15 years, several studies have documented transfer of mitochondria from different types of MSCs to aberrant cells via TNTs. In Table 2, we have summarized these studies, most of which have shown the involvement of oxidative stress, mitochondrial stress, ROS and/or apoptotic stress in the biogenesis of TNTs or cell-to-cell transfer via TNTs. Transfer of mtDNA and healthy mitochondria from MSCs via TNTs can be a potential remedy.

**Table 2:** Transfer of mitochondria from different types of MSCs to aberrant disease models via TNTs

Donor cell/source	Disease model	Experimental model used in the study	Mechanism/signals involve in TNT formation	Results
MSCs derived from human adipose tissues	Oxidative stress	In vitro: MSCs were subjected to hydrogen peroxide, *N*-acetyl-l-cysteine, and l-ascorbic acid 2-phosphate	Oxidative stress and mitochondrial dysfunction	Antioxidants increased the mitochondrial mass and respiratory capacity [89]
Rat bone marrow MSCs	Cardiovascular	In vitro model of ischemia–reperfusion injury	Hypoxia in the target cells	Decrease in the rate of apoptosis in H9c2s [90]
Wharton jelly mMSCs	MELAS patients (Mitochondrial Encephalopathy, Lactic Acidosis, and Stroke-like episodes)	In vitro: rotenone treated stressed Human MELAS fibroblasts	Eliminates mt.3243A>G mutation burden	Rescues bioenergetics of mitochondria in rotenone-stressed MELAS fibroblast [91]
Wharton jelly mMSCs	MERRF (myoclonus epilepsy associated with ragged-red fibers)	In vitro: increased ROS levels and oxidative stress	Eliminates mt.3243A>G mutation burden	Rescues bioenergetics of mitochondria and alleviates ROS levels in MERRF model [92]
Human iPSC-MSCs	Oxidative stress	In vitro: rotenone was used in the corneal epithelial cells In vivo: sodium hydroxide induced a corneal alkaline burn in the rabbit model	Oxidative stress and mitochondrial dysfunction	In vitro: protection against rotenone oxidative stress In vivo: beneficial effects for corneal wound recovery [93]
Human bone marrow MSCs	Lung injury	In vitro: secreted medium from the macrophages that were exposed to IL-13 was used to treat the mouse bronchial epithelial cells In vivo: epithelial injury and allergic airway inflammation was induced by rotenone treatment in a mouse model	Epithelial mitochondrial dysfunction	A higher mitochondrial transfer was seen in the overexpressed Miro1 MSCs [9]
Mouse and human bone marrow MSCs	Lung injury	In vivo: mouse acute lung injury model	Acute lung injury caused dysfunction in the mitochondria	Shielding effects were observed by the mitochondrial transfer via TNTs [83]
Human iPSC-derived MSC	Mitochondria damage	In vitro: PC12 cells were exposed to the CoCl2 (a chemical inducer of hypoxia inducible factor-1)	ROS	Reduction in mitochondrial dysfunction was detected [94]
Mesenchymal Multipotent stromal cells (MMSCs)	Kidney transplantation	In vitro: rat renal tubular cells	Induction of differentiation	A two-directional exchange of cytoplasmic content was seen [95]
Human bone marrow MSCs	Inflammatory disease	In vitro: human vascular smooth muscle cells	Mitochondrial dysfunction in vascular smooth muscle cells	There was an enhanced MSC proliferation but not differentiation was detected [96]
Rat bone marrow MSCs	Inflammation	In vitro: rat nucleus pulposus cells subjected to IL-1β	Excessive apoptosis	There was a reduction in apoptosis in the direct co-culture method [97]
MMSCs	Post-ischemic stroke	In vitro: post-ischemic model in rat cortical neurons	Post-ischemic stress	Better rehabilitation after stroke [98]
MMSCs	Ischemic model	In vitro: ROS elevated ischemic model in neural cells, and astrocytes	Elevated ROS levels	Restored bioenergetics and stimulated proliferation [99]
BM-MSCs	Spinal cord injury	In vitro: oxygen-glucose deprived (OGD) injured VSC4.1 motor neurons or primary cortical neurons	Oxygen–glucose deprivation	Improved bioenergetics and recovery of OGD and spinal cord injury models [100]
BM-MSCs	Acute respiratory distress syndrome (ARDS)	In vitro: monocyte-derived macrophages	Stress due to E. coli infection	Enhanced phagocytosis [101]
BM-MSCs	Acute respiratory distress syndrome (ARDS)	In vitro: lipopolysaccharide (LPS) induced monocyte-derived macrophages	LPS-induced stress	Enhanced phagocytosis [102]
BM-MSCs,	Myocardial infarction	In vitro: ischemic H9c2 cardiomyoblasts	Oxygen–glucose deprivation	Increased survival rate of cardio-myoblasts [103]
MSCs,	Cardiomyopathy	In vitro: cardiomyocytes	LPS-induced stress	Enhancement of myocardioblast functions due to bioenergetics stimulus [104]
iPSCs-MSCs	Cardiomyopathy	In vitro: cardiomyocytes	Cardiomyopathy induced by anthracycline	Rescued of cardiomyopathy by transferring of Miro1 and TNFαip2 [105]
iPSCs-MSCs	Chronic obstructive pulmonary disease (COPD)	In vitro: bronchial epithelial cells	Cigarette smoking (CS) induced COPD	Rescued CS induced mitochondrial damage [106]

However, a deeper understanding is needed to implement the transfer of mitochondria as a therapy, and focus should be given to unravelling various stress signals that could affect transcellular trafficking of mitochondria via TNTs, both in diseases and in rejuvenation [3, 107, 108].

## Association of tunnelling nanotubes with oxidative stress, apoptosis, and mitochondrial homeostasis

Mitochondria play an important role in oxidative phosphorylation, aerobic metabolism, calcium signalling, and apoptosis [109]. Mitochondrial dysfunction-related oxidative stress is associated with diseases such as cardiomyopathy, ischemic heart diseases, lung disorders, brain injury, stroke, and neurodegenerative diseases like Alzheimer’s and Parkinson’s disease. Exchange of mtDNA between cells via transfer of mitochondria could modulate respiration and cell cycle arrest. Levels and homoplasmic polymorphism of mtDNA regulate mtDNA-processing enzymes, replication, and transcription of mtDNA and respiratory complexes. Dysfunction of these processes can result in aberrant mitochondria with formation of ROS and also cell cycle arrest due to impaired function of the respiration-linked enzyme dihydroorotate dehydrogenase [110]. Melanoma cancer cells devoid of mtDNA injected in to syngeneic C57BL/6N^*su9-DsRed2*^ mice expressed with red fluorescent mitochondrial protein can recover to form tumours after import of mtDNA by acquiring whole mitochondria from neighbouring healthy cells [81]. Oxidative stress and ROS promote the biogenesis of TNTs in several pathological conditions [54]. Hydrogen peroxide (H_2_O_2_) treatment in the primary hippocampal rat astrocytes and neurons promotes the biogenesis of TNTs, at the same time the induced cellular stress activates tumour suppressor protein p53 [12]. However, later studies were reported that p53 is not the key element for TNT formation, and the effect of H_2_O_2_ on TNTs is cell type-specific [111].

The crucial role of intercellular, horizontal transfer of mitochondria demonstrated recently under various pathophysiological conditions, primarily in rescuing tumourigenesis and bioenergetic deficiencies. Tan et al. [108] have shown that the mtDNA-deficient cells acquired functional mitochondrial genome from the surrounding tumour microenvironment or MSCs to regulate many factors related to mitochondrial respiration. In cancer cells, delaying apoptosis resulted in the restoration of cell survival and enhancement of tumourigenicity or metastasis. MSCs from different sources exert different rescue capacities against aerobic respiration ability and postpone apoptosis of the recipient cells [23, 107]. It is possible that paracrine factors related to oxidative stress and/or ROS sent from stressed cells trigger MSCs to make cellular bridges via TNT structures for transferring mitochondria.

The role of TNTs in rescue from apoptotic cell death has also been demonstrated in neuronal cells [25]. This study showed that PC12 cells that were treated with UV light were rescued by non-cancer cells by transfer of mitochondria via TNT-like structures when compared with untreated cells. The UV treated cells that had lost cytochrome C formed TNTs but did not enter the apoptotic cascade. The study suggests that transfer of mitochondria from healthy cells via TNTs reverses the cellular stress in early stage of apoptosis. A recent work [112] has shown that α-synuclein protofibril-induced defects in cellular degradation machineries in microglia enhance cell to cell networks via TNTs to transfer the burden of proteotoxic aggregates to neighbouring cells. The study has also shown that mitochondrial shuffling and sharing of proteotoxic burdens via TNTs alleviate ROS levels and rescue cells from ROS-induced apoptosis.

## Rho GTPase related signals counteract apoptosis via tunnelling nanotubes

TNTs mediate direct intercellular transport between neighbouring cells and, structurally, they are open-ended membrane actin conduits. Thus, modulation of membrane and cytoskeleton dynamics may play a major role in the biogenesis of TNTs. Several studies have shown that actin-depolymerizing agents such as cytochalasin B and latrunculin B inhibit TNT formation [1, 113]. The master regulators of the cytoskeleton, Rho family of GTPases (Rac1, Cdc42, and RhoA), are implicated in TNT formation by many studies [13]. The localized control of Rho GTPase regulators, the GTPase activating proteins (GAPs) and guanine nucleotide exchange factors 42 (GEFs) have been proposed to play a role in TNT assembly. One study [114] in immune cells has reported that Cdc42 and Rac1, and their respective effector molecules WASP and WAVE2, are involved in the biogenesis of TNTs by modulating actin polymerization via the Arp2/3 complex. Using FRET-based biosensors, the study has demonstrated that Rac1 stays distributed throughout the TNT structures, while Cdc42 is involved in initiating the biogenesis of TNTs. Transfer of oncogenic KRAS promotes formation of TNTs by regulating the ERK pathway in colorectal cancer. It is thus important to note that Rho GTPase-regulated ERK signalling pathway controls the expression of pro-survival or anti-apoptotic Bcl-2 family of proteins [55].

Two actin regulators downstream of Rho GTPases, βCamKII and cofilin, have recently been demonstrated to play a role in the biogenesis of TNTs. Cross-talk between the signalling cascades of Rho GTPases with the actin regulatory molecules βCamKII, cofilin and Arp2/3 is well documented in the early development of the dendritic spine [115]. Vargas et al. [116], showed that stability of TNTs depends on the activation of the Wnt/Ca^2+^ signal-dependent modulation of βCamKII in the CAD (mouse catecholaminergic neuronal cell line) cells and primary neurons. The actin-binding ability of the protein is modulated by phosphorylation of βCamKII [117]. Inactivation of cofilin by the RNA-binding protein nucleolin induces TNT biogenesis [118]. In addition, the alphaherpesvirus-induced biogenesis of TNTs depends on the US3 protein kinase-mediated activation of p21-activated kinases (PAKs) apparently by activation of Cdc42/Rac1 and Rho signalling axis, within a poorly understood complex mechanism [32]. PAK kinases are considered primarily the effector of the Rho family GTPases Cdc42 and Rac1. Additionally, studies have shown that PAK1 inhibitor IPA-3 attenuates alpha herpes virus-induced TNT-like membrane actin projections [32, 119]. PAK2 has also been reported in HIV-1 Nef protein-mediated TNT formation [120].

In our recent study, we have observed that Alzheimer’s pathogenesis, the amyloid-β oligomers internalize via PAK1 dependent actin mediated endocytic pathway, and the internalization process promotes formation of TNT-like structures and direct cell-to-cell transfer of oligomers in neuronal cells [39]. The study has also shown colocalization of activated PAK1 with f-actin throughout the TNT network.

Conversely, the Cdc42/IRSp53/VASP system plays a role in the filopodia-promoting network, being negatively correlated with formation of TNTs in neuronal cells [121]. Recently, another study has reported that Arp2/3 negatively regulates biogenesis of TNTs in CAD cells [5]. Another recent study [112] has shown that inhibition of ROCK (using chemical inhibitor Y-27632), a downstream signalling molecule of Rho/Rac/Cdc42, promotes biogenesis of TNTs. The study has indicated that ROCK inhibition promotes TNT formation via Myosin II regulated f-actin modulation. Altogether, these studies suggest a complex regulatory mechanism of Rho GTPases in TNT biogenesis. A further recent report [118], has also shown that M-sec regulated exocyst complex needs to function together with actin polymerization by inhibiting activity of cofilin in the biogenesis of TNTs in multiple mammalian cellular models. The study suggests that in addition to actin polymerization, M-Sec-dependent plasma membrane (PM) re-modelling is a necessary step in formation of TNTs.

The rescue capacity of MSCs mediated via TNTs correlates with the Miro-1 expression, as shown for the transfer capacity of mitochondria from MSCs to stressed alveolar epithelial cells via TNTs [9]. Miro-1 and -2 belong to a class of novel Rho-GTPase, amino acid sequence revealed GTPases domain homolog to the classical Rho-GTPases in the N-terminal part of the protein [16]. Interestingly, Miro proteins lack the membrane-binding motif CAAX in their C-terminal domains, unlike small GTPases but contain a second GTP-binding domain without homology to typical Rho-GTPases [15]. Studies have shown that overexpression of Miro-1 protein leads to an increase in the mitochondrial transfer capacity and, hence, there is a decrease in the apoptosis level and mitochondrial ROS production, and alleviation of respiratory dysfunction [122]. A recent study has shown that the monooxygenase domain of MICAL2PV, a spliced isoform product of the neuronal guidance gene MICAL2, interacts with Miro-2, inhibiting TNT formation by depolymerization of f-actin. MICAL2PV plays crucial role in cell survival and down-regulation of MICAL2PV, and protect lung cancer cells treated with chemotherapeutic drugs [123].

## Rho GTPases in cell surface dynamics and TNT biogenesis

Several cytoskeleton remodelling signals are correlated with cell surface dynamics and PM remodelling [124]. Small GTPases Arf and Rab regulate exocytosis of specific vesicles to discrete sites of the PM. Rho GTPases and their regulatory factors contribute to the process by modulating the tethering and subsequent fusion of exocytic vesicles. One study [125], showed that formation of TNTs is regulated by the exocyst complex protein M-Sec in HeLa cells, which is involved in exosome fusion and membrane expansion. The exocyst complex contributes to PM recruitment of the actin remodelling proteins Ral-GTPase and filamin to promote TNTs. The regulatory molecules associated with the recycling of endocytic vesicles and vesicle trafficking, which regulates PM surface dynamics, have also been implicated by several studies in the biogenesis of TNTs. Rab class of small GTPases, Rab8a, Rab11a, and Rab35 are implicated in TNT formation by regulating membrane recycling in neuronal and cancer cells [126, 127]. Rab35-GTP, ACAP2, ARF6-GDP, and EHD1 promote TNT formation in a cascade-like manner in neuronal cells. It may therefore be that modulation of cytoskeleton remodelling via actin polymerization signalling cascades is linked to cell membrane surface dynamics to induce formation of membrane actin-derived TNTs.

## Rho GTPases in cell cycle progression, apoptosis, and TNT biogenesis

RhoA, Rac1, and Cdc42 are the most studied typical Rho GTPases, not only involved in the regulation of distinct actin cytoskeleton and PM structures, they are also interlinked via complex molecular signalling events to regulate cell cycle progressions and apoptosis [128]. Rac1-regulated oxidase was reported to modulate acute cellular necrosis, apoptosis, and acute inflammatory response in hepatic ischemia. Rac1-induced production of ROS by an NADPH oxidase was also reported in both phagocytic and non-phagocytic cells [129]. Rac1 can also activate signalling downstream of NFκB, PAK, and ERK by ROS-mediated pathways in neuronal cells to counteract apoptosis. Neuronal cells have limited regenerative capability, and continuous ‘fitness’ of these cells is vital; these cells possess intrinsic competence to attenuate apoptosis [130]. Instead, apoptosis due to elevated stress/ROS levels in neuronal cells may induce formation of TNTs to ameliorate cellular stress [54]. In cancer cells, Rac1-mediated MAPK/ERK and Akt signalling involves the upregulation of the pro-survival or anti-apoptotic Bcl-2 family of proteins [131]. The pro-survival signalling of MAPK/ERK involving formation of TNTs occurs in various cancer cells [132], and TNTs promote cell proliferation and cancer malignancy levels [48]. In addition, TNTs are involved in transfer of apoptosis regulators from healthy cells to diseased cells. Several studies have also shown that the pro-apoptotic Fas ligand is transferred via TNTs to T lymphocytes to induce cell death [133, 134].

## Conclusions

The discovery of TNTs in 2004, opened up a novel mechanism of long-range intercellular communication. TNTs are actin-membrane conduits, thereby, actin regulation together with dynamic PM modulatory cellular events play major roles in their biogenesis. The complex functions of Rho GTPase signalling cascades have been implicated by several studies in TNT biogenesis. However, some contradictions exist in the literature and there may be some variability in TNT regulation in different cell types. Moreover, discrepancies also exist in the definition of supercellularity of TNT structures in different studies. It is challenging to resolve TNTs and TMs in ex vivo organoid models or in vivo animal models. Detection methods using advanced imaging tools or exclusive markers need to be explored to make advancement in the field.

**Fig. 1:**
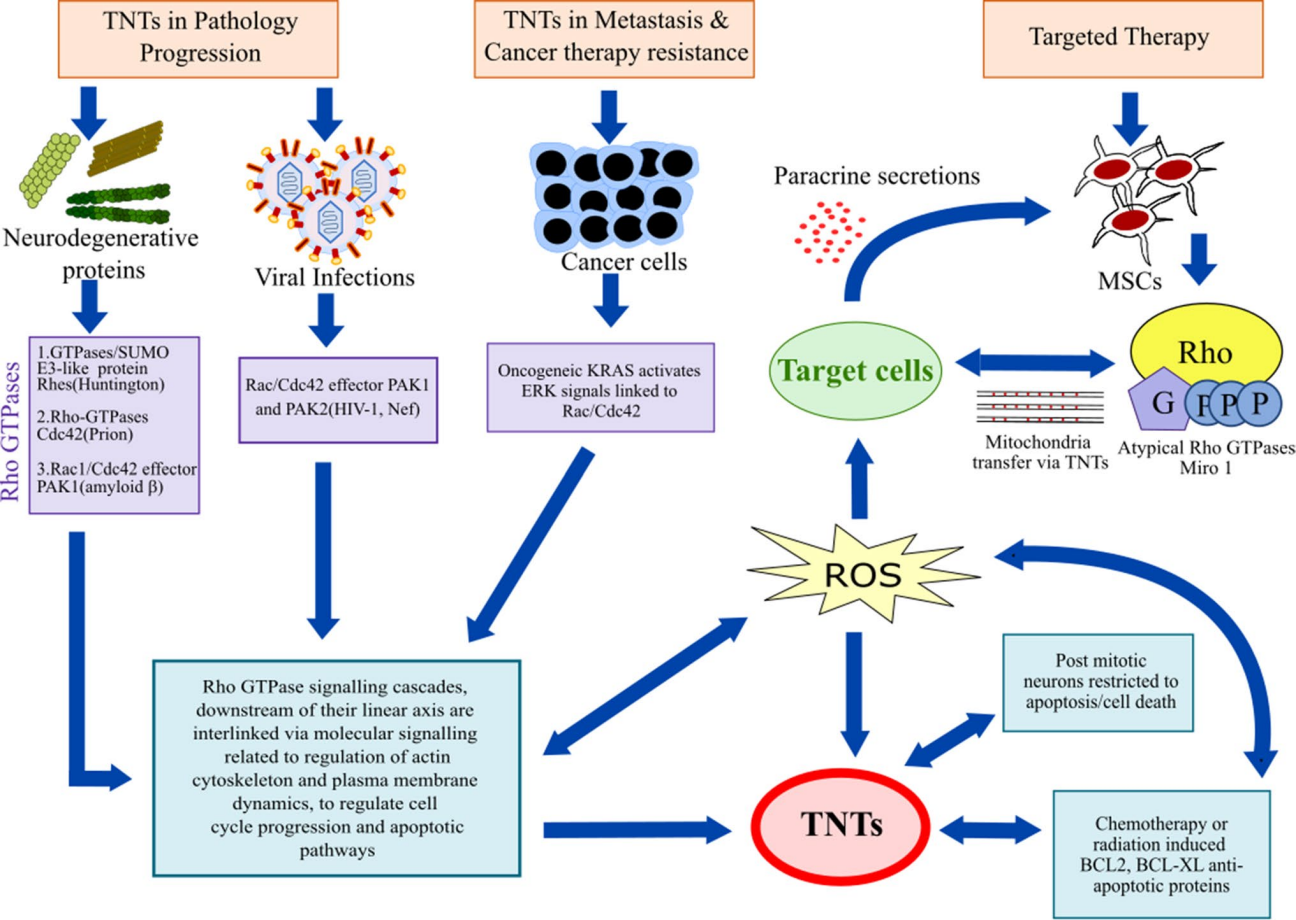
Schematic summary of TNT studies indicating the involvement of Rho GTPase signalling cascades in the biogenesis of TNTs by modulating actin cytoskeleton proteins, PM dynamics and potentially alleviating cellular or apoptotic stress

Rho GTPase signalling cascades, that are not only related to the regulation of distinct actin cytoskeleton and PM dynamics, downstream of their linear axis are interlinked via complex molecular signalling events to regulate cell cycle progression and apoptosis (Fig. 1) [128, 131]. Direct cell-to-cell transfer of organelles or cargo via TNTs has emerged as an important mechanism for maintaining cellular homeostasis, and this process has been implicated in disease spread and disease resistance [1]. The widespread association of oxidative stress, apoptosis, mitochondrial homeostasis, and mitochondrial heteroplasmy with the biogenesis of TNTs has been established by several studies [1, 113]. Cell types that possess an inherent mechanism to resist apoptosis, such as neuronal cells and cancer cells, promote the biogenesis of TNTs possibly to maintain cell survival under pathological stress. Some studies for example [10], have shown that ROS and apoptotic stress promotes the biogenesis of TNTs, however, the molecular events associated with apoptosis signalling or oxidative stresses are not the primary regulatory elements. Biogenesis of TNTs increases the survival of cancer cells treated with chemotherapy, radiotherapy, UV radiation, and laser-induced phototoxicity. MSCs rescue cells from apoptotic death triggered by oxidative stress or mitochondrial heteroplasmy. Therefore, MSC-mediated transfer of mitochondria could have therapeutic potential, for example, by promoting wound healing in response to mitochondrial import [135]. On the other hand, transfer of healthy mitochondria rescues ROS-induced apoptosis in cancer cells and promotes cancer malignancies. It is unclear to what extent damage to mitochondria triggers the formation of TNTs. Do damaged recipient cells actively form TNTs to healthy neighbouring cells? If not then, what signal triggers healthy cells to make direct connections via TNTs to transfer mitochondria. Several articles have shown that the atypical Rho GTPases, Miro 1 and Miro 2, play significant roles in cell to cell transfer of mitochondria from MSCs. Classical Rho GTPases are implicated in other cell types, such as neuronal cells, immune cells and in the transfer of virus spreading. Structurally and functionally these two types of Rho GTPases are distinct, although they do share several homologous domains and may have overlapping functions in TNT signalling pathways. Thus, future studies are required to investigate the emerging role of Rho GTPase signalling cascades in TNT biogenesis and in the formation of supercellular structures with potential importance in maintaining tissue homeostasis and pathophysiological conditions.

## Abbreviations

ALL: Acute lymphoblastic leukemia
AML: Acute myeloid leukemia
BMSC: Bone marrow stromal cells
CAD: Catecholaminergic neuronal cells
EVs: Extracellular vesicles
ERK: Extracellular signal-regulated kinase
GBM: Glioblastoma Multiforme
HIV: Human immunodeficiency virus
HUVEC: Human umbilical vein endothelial cells
iPSC: Induced pluripotent stem cells
MIRO: Mitochondrial Rho GTPase
MSCs: Mesenchymal stem cells
mtDNA: Mitochondrial DNA
PAK1: P21-activated kinase 1
PC12: Pheochromocytoma cells
Rho: Ras homologous protein
ROS: Reactive oxygen species
TASC: Tumour activated stromal cells
TNTs: Tunnelling nanotubes
TMs: Tumour microtubes
UV: Ultraviolet
